# Context-dependent benefits of forest soil addition on Aleppo pine seedling performance under drought and grass competition

**DOI:** 10.1007/s00572-024-01151-x

**Published:** 2024-05-18

**Authors:** Lior Herol, Mor Avidar, Shahar Yirmiahu, Yair Yehoshua Zach, Tamir Klein, Hagai Shemesh, Stav Livne-Luzon

**Affiliations:** 1 Department of Environmental Sciences, Tel-Hai College, Qiryat Shemona, Israel; 2 Department of Plant and Environmental Sciences, Weizmann Institute of Science, Rehovot, Israel

**Keywords:** Branching, Climate change, Competition, Drought, Ecology, Ectomycorrhiza, *Geopora*, Soil inoculation

## Abstract

Seedling establishment under natural conditions is limited by numerous interacting factors. Here, we tested the combined effects of drought, herbaceous competition, and ectomycorrhizal inoculation on the performance of Aleppo pine seedlings grown in a net-house. The roots of all pine seedlings were strongly dominated by *Geopora*, a fungal genus known to colonize seedlings in dry habitats. Ectomycorrhizal fungi (EMF) inoculum significantly increased seedling height, biomass, and the number of side branches. However, under either competition or drought, the positive effect of EMF on seedling biomass and height was greatly reduced, while the effect on shoot branching was maintained. Further, under a combination of drought and competition, EMF had no influence on either plant growth or shape. The discrepancy in pine performance across treatments highlights the complexity of benefits provided to seedlings by EMF under ecologically relevant settings.

**Supplementary Information:**

The online version contains supplementary material available at 10.1007/s00572-024-01151-x.

## Introduction

Forests are one of the biomes most affected by the recent climatic changes, expressed in an increase in fire frequency, tree mortality, and slower forest regeneration (Kowaljow et al. 2019). This recent increase in mature tree mortality highlights the role of seedling establishment in supporting the sustainability of forests in the future (Whitmore 1998). However, seedling establishment is often limited by numerous biotic (Gorchov and Trisel 2003; De La Cruz et al. 2008) and abiotic (Alvarez‐Aquino et al. 2004) environmental factors. Because of their small size and relatively slow growth, seedlings are often at a disadvantage when competing with mature trees (Dickie et al. 2005) or herbaceous vegetation (Van Der Waal et al. 2009). This disadvantage, which results from their limited access to aboveground (light) and belowground (water and nutrients) resources, can hamper seedling growth and reduce their resilience to droughts (Cavender-Bares and Bazzaz 2000; Pozner et al. 2022) and competition (Fetene 2003).

The survival, establishment and growth of many tree species depends on ectomycorrhizal interactions (Rudgers et al. 2007; Smith and Read 2010; Peay 2016). However, the extent to which plants experiencing stressful conditions can benefit from ectomycorrhizal interactions is much less clear (Boyle and Hellenbrand 1991). Plant-fungal competition over shared resources or the ability of one or both partners to cope with environmental stress could limit the value of the interaction under conditions of inter-plant competition or drought.

By increasing the surface area of the seedlings root system, ectomycorrhizal fungi (EMF) allow the roots to absorb minerals and water from a wider surface (Rousseau et al. 1994). Under drought conditions, ectomycorrhizal mycelium can benefit trees by extracting inaccessible water from micro-crevasses within soil particles (Bornyasz et al. 2005) as well as the transport of water over larger soil volumes (Duddridge et al. 1980). Wang et al. (2021), show that under water limited conditions, ectomycorrhizal fungi can increase stomatal conductance and photosynthetic rates, which lead to an increase in plant growth (i.e., height and biomass increase), while reducing their mortality rate. However, all these plant benefits are dependent on the EMF ability to survive and perform under stress (Di Pietro et al. 2007). Because both the fungi and the seedling require water, it is not clear whether the ectomycorrhizal interaction can remain beneficial to the seedling under severe drought conditions. Moreover, some specific plant physiological responses to drought like increased suberisation (Brunner et al. 2015) and reduced fine root allocation (Joseph et al. 2020) may even hinder the interaction for some EMF species.

While drought is a pivotal stressor in many ecological systems, it is often combined and possibly enhanced by inter-plant competition (Kaisermann et al. 2017). Unlike the effects of EMF on plant ability to withstand drought, which did receive considerable attention in the literature (Kennedy and Peay 2007; Lilleskov et al. 2009; Lehto and Zwiazek 2011; Kipfer et al. 2012; Nickel et al. 2018; Sebastiana et al. 2018; Xu and Zwiazek 2020; Querejeta et al. 2021; Wang et al. 2021), the influence of EMF on a seedling’s ability to cope with other plant competitors was less studied. Only a few studies tested the effect of EMF on inter-specific plant competition (Pedersen et al. 1999; Shi et al. 2017; Peay 2018; Van Nuland et al. 2023). In these studies, EMF inoculation has been shown to positively affect the competitive ability of it hosts. These effects seem to depend on the nature of the competitor (Shi et al. 2017), the nutrients available in the soil (Pedersen et al. 1999; Van Nuland et al. 2023) and the timing of the inoculation (Peay 2018). To the best of our knowledge, no manipulative studies have been conducted on the interaction between drought, inter-specific plant competition and EMF in their effect on seedling performance.

In this study we tested whether EMF provide an advantage to Aleppo pine (*Pinus halepensis* Miller) seedlings experiencing drought, competition with an annual grass, or both. Mediterranean tree species are prone to relatively frequent environmental changes such as droughts (Petit et al. 2005; Garcia de Jalon et al. 2020) which could be exacerbated by competition with annual vegetation. Aleppo pine is the most common forest tree species around the Mediterranean basin (Ne'eman and Osem 2021), is well adapted to local conditions (Klein et al. 2011; Voltas et al. 2018; Patsiou et al. 2020) and highly dependent on EMF (Livne-Luzon et al. 2017, Avital et al. 2022; Cahanovitc et al. 2022). We hypothesized that the relative advantage that the EMF can provide to the pine seedlings will increase under drought and/or competitive conditions since both stressors will minimize the ability of the plant to acquire soil resources independently. Under the combination of both stressors the importance of EMF presence should be more pronounced.

## Materials and methods

### Experiment overview

We experimentally manipulated the growth conditions of Aleppo pine seedlings. Specifically, seedlings had varying levels of ectomycorrhizal inoculum (with/without), water conditions (full irrigation/ drought) and grass competition (with/without). The complete crossing of these three factors created 8 different groups. Each group was replicated 28 times (2 water × 2 EMF × 2 competition × 28 replications = 224 pots). Six spare plants were added as additional replicates forming an incomplete block resulting in a total of 230 pots. The experiment was initiated during February 23 -27, 2020 and was conducted for six months.

### Growth conditions

The experiment was conducted under natural daylight (12–13 h) and annual ambient air temperatures (21.6 ± 6 °C) in a net-house in Tel-Hai College, Israel (N 35°34′41"E 11°33′14"), from the 23rd of February to the 23rd of August.

### Potting material

Grassland soil and sand were used as potting material. The soil was collected in herbaceous Mediterranean grassland, lacking EM host plants—according to previous experiments (Livne-Luzon et al. 2017, Livne-Luzon et al. 2021) from a field near Tel-Hai College (33°13′56"N 35°34′48"E). The potting material was mixed (50% sand, 50% grassland soil) using an electric cement mixer. The potting material was analyzed using a MASTERSIZER 3000. Soil texture was 71% sand, 22% silt, and 7% clay. The pots (4 L) were filled with the mixture. The grassland soil used for potting material in this study was used in a few previous studies (Livne-Luzon et al. 2017, Livne-Luzon et al. 2021) and DNA sequencing revealed negligible reads of only a single EMF taxa that did not appear on seedlings roots.

### Ectomycorrhizal inoculum

In order to increase the study’s ecological validity, we chose to add natural forest soil which includes a diverse EMF community (Livne-Luzon et al. 2017, Avital et al. 2022). Forest soil was collected from four different locations (Table S1). All forest soils were collected underneath Aleppo pine trees approximately 50 years old. At every location, soil was sampled at four locations spaced 1–2 m apart. The depth of the taken soil was a few centimeters below the surface in order not to collect the remnants of the organic matter. All the soils collected were mixed and sieved (2 mm). The forest soil (100 ml) was mixed into the EMF treatment pots. To maintain an equal volume, 100 ml of potting material (50% sand, 50% grassland soil) was mixed into the pots without the EMF.

### Plant material

Pinecones were collected three days before the beginning of the experiment from five different individual pine trees growing in a 1000 m^2^ area (Table S1, location 1). The pinecones were placed in an oven to release the seeds from the cones (15 min, at 80 °C). Four seeds were sown in each pot, and the pots were arranged in blocks (8 pots per block). All the pots were irrigated with a computerized irrigation system (20 min in the morning every day, at a capacity of one liter per hour).

### Competition stress

On the 4th day of the experiment, *Hordeum spontaneous* seeds were sown in the pots experiencing competition (4 in every pot). *Hordeum spontaneous* (K. Koch) is a fast-growing grass common in the southern and eastern Mediterranean. Forty days after sowing, both *Hordeum spontaneous* and *Pinus halepensis* were thinned down to one per pot. Thinning was executed by pulling the seedlings with their roots, out of the soil.

### Drought conditions

Sixty-two days after sowing, the pots in the drought treatment were disconnected from the irrigation system, while the control treatment remained connected, maintaining a saturation condition. The pots were irrigated manually to create drought stress (Table S2). The weight of the water in saturation was calculated by reducing the weight of the dry pot (the pot's mass after drying in the oven—4.375 kg), from the weight of a pot at water content at saturation (5.75 kg). The difference is the weight of the water in saturation (1.375 kg). In the drought treatment, the pots were maintained at 10% from saturation level (0.1375 + 4.375 = 4.52). The amount and frequency of the irrigation were determined in accordance with the average weight of the pots. The changes in the weight of the pot were caused because of changes in the weather during the experiment as well as plant growth. Ten pots were weighed prior to every irrigation event and 40 pots (10 pots from each treatment) were weighed four times during the experiment (Table S2).

### Morphological measurements

One hundred and sixty-one days after sowing, the pines' height and the number of side branches bifurcating out of the main stem were measured. The purpose of these measurements was to characterize morphological differences between the different treatments. We standardized the branch number by dividing the number of branches by the total pine biomass. This was done to obtain an index of the number of branches, irrespective of the size of the pine.

### Harvesting protocol

One hundred and seventy-eight days after sowing, plants were harvested by block. Plants were removed intact from the pots and washed gently under tap water and the roots and shoots of both inoculated and uninoculated plants were separated. The roots of all plants, regardless of inoculation treatment, were scanned visually, with the use of a dissecting microscope when needed, for colonized root-tips. Additionally, for each seedling, the number of colonized and uncolonized root tips was recorded for five representative roots. This enabled us to calculate the percent of colonization for each root (the number of colonized root tips divided by the total number of root tips on that root). We later calculated the average percent colonization for the five roots of each seedling. All root tips of the same individual, either colonized or suspected of being colonized, were removed using sterilized forceps, inserted into a 1.5 ml Eppendorf tube added with 300 µl CTAB buffer, and stored in a -20°C freezer until DNA extraction. Both roots and shoots were placed separately in the oven (60 C°, for 3 days). Shoot and root mass were measured using an analytical scale (Radwag, AS 220.R2, Radom, Poland). Out of the initial 230 pines, 17 pines died during the experiment and 2 pines had extreme biomass values (z = 3.63; z = 4.12) and have been excluded (with no qualitative changes to the results; Fig. S2-4, Table S3) from further morphological analyses.

### Needles nitrogen quantification

During the analysis of the biomass and amplicon sequencing data we developed a post-hoc hypothesis regarding the role of competition for nitrogen (see discussion). We then decided to quantify the nitrogen content of the pine needles. The nitrogen content was estimated only for five experimental blocks (a total of 40 pines). In order to choose representative blocks, standard Z scores were calculated for the mean total pine biomass of every block. The 5 blocks with the smallest absolute Z scores were used in the analysis. Two hundred mg of needles were taken from each seedling. In small pines with less than 200 mg, all needles were used (156.6 ± 49 mg for all pines combined). Nitrogen content was quantified following the Kjeldahl method using Kjeltec^TM^ 8100 (Foss, Denmark). Nitrogen content was then divided by the needle biomass to attain the percent of nitrogen in the tissue.

### Molecular identification of fungal species

DNA was extracted from the roots of 76 different pine seedlings following the methods of (Livne-Luzon et al. 2017). The 76 pine seedlings were selected from all eight treatments. The uninoculated treatments were only replicated 4 times because we were unable to visually spot EMF colonization on their roots and didn’t expect the roots of these pines to be significantly colonized with ectomycorrhizal fungi (see Table S4 for the distribution of the samples between treatments). Briefly, frozen root tips were bead beaten (at least 2 × 30 s at 4000 rounds per minute till fine powder was achieved), and DNA was extracted from each root tip sample following a modified version of the QIAGEN (Valencia, CA, USA) DNA easy Blood and Tissue Kit. Barcoded amplicon sequencing of the fungal ITS2 region was performed on a MiSeq platform (Illumina, San Diego, CA, USA). A two step protocol for library preparation was performed according to Straussman lab (Nejman et al. 2020), with several modifications; First PCR reactions were performed using KAPA HiFi HotStart ReadyMix DNA polymerase (Hoffmann-La Roch, Basel, Switzerland) in 50 µl reaction volumes with 5 µl DNA extract, and 1 µl of every primer 5.8S-Fun (5′- AACTTTYRRCAAYGGATCWCT) (Taylor et al. 2016) and RD2-ITS4Fun (5′AGACGTGTGCTCTTCCGATCT-AGCCTCCGCTTATTGATATGCTTAART). The reverse primer consisted of the ITS4-Fun primer (Taylor et al. 2016) with the linker adapter RD2. PCR reactions were performed as follows: initial 2 min at 98 °C followed by 35 cycles of 10 s 98 °C, 15 s 55 °C, and 35 s 72 °C final cycle with 5 min 72 °C. Second PCR reactions were performed using the same DNA polymerase in 50 µl reaction volumes with 1/10 (5 µl) of the first PCR reaction, and 1 µl of every primer P5-rd1-5.8S-Fun (5′- AATGATACGGCGACCACCGAGATCT-ACACTCTTTCCCTACACGACGCTCTTCCGATCT-AACTTTYRRCAAYGGATCWCT) and RD2-Barcode (5′ AGACGTGTGCTCTTCCGATCT-BARCODE). The forward primer consisted of the adaptor p5 the linker RD1 and the primer 5.8S-Fun. The reverse primer consisted of the adapter RD2 and the individual barcode. PCR reactions were performed similar to the first PCR but with only 6 cycles. PCRs were cleaned using Qiaquick PCR purification kit (Qiagen, Hilden, Germany), quantified fluorescently with the Qubit dsDNA HS kit (Life Technologies Inc., Gaithersburg, MD, USA). Libraries were quality checked for concentration and amplicon size using the Agilent 2100 Bioanalyzer (Agilent Technologies, Santa Clara, CA, USA) and size selected with AMPure magnetic beads (Beckman Coulter Inc., Brea, CA, USA). We sequenced all the samples in one amplicon using Illumina MiSeq technology with 300 bp paired-end reads (PE300_V3) in the Grand Israel National Center for Personalized Medicine (Weizmann institute of science, Rehovot, Israel).

### Bioinformatics

We used R (R Core Team 2021, version 4.0.3) and the R-Studio for bioinformatics and statistical analysis. Raw sequences were demultiplexed and adapters together with barcodes were removed for 76 root samples (Water × Ectomycorrhiza × Competition). The sequences were analyzed using the amplicon sequencing dada2 package v. 1.7.9 in R (Callahan et al. 2016). In summary, sequences were quality-filtered and trimmed. We only used sequences longer than 50 bases with a mean number of expected errors below 2 (maxN = 0, maxEE = c(2,5) minLen = 50 truncQ = 2). Paired-end sequences were merged using the MergePairs function. We then applied a dereplication procedure on each sample independently, using derepFastq function. Finally, all files were combined in one single Fasta file to obtain a single amplicon sequence variant (ASV) data file. We removed singletons (minuniquesize = 2) and de novo chimera sequences using removeBimeraDenovo function against the reference database (UNITE/UCHIME reference datasets v.7.2). Sequences were then clustered, and taxonomic assignment (id = 0.98) was done against the UNITE database. Non-fungal ASVs were removed. FUNguild was then used to parse ASVs into ecological guilds (Nguyen et al. 2016); we then filtered the table to include only highly probable EMF genera. Due to the low colonization rates of the young seedlings, the average sequence abundance was very low (the mean sequence abundance per sample was 1142 ± 1516, for *Geopora* 1167 ± 1507) and the total number of putative EMF ASVs (16) was low as well.

### Statistical analysis

We used general linear mixed models, using a fully factorial design, to account for differences in the pine's total, shoot and root dry biomasses, pine height, root EMF colonization percents, branch density and the relative abundance of the most dominant EMF taxa (i.e., *Geopora).* The following explanatory variables were included as fixed factors: EMF inoculation, water and competition treatments as well as their interactions. The experimental blocks were included in the model while allowing for random slopes and intercepts for each fixed factor. Due to high variation and skewedness of the data, the branch density, and the sequence abundance of *Geopora* were log transformed prior to analysis. Because all the interactions in the data were ordinal (none-crossover), we report both the main effects and the interactions. Figures were generated using R packages ggplot2 (version 3.3). Illustrations were created with BioRender.com.

## Results

For comprehensive details regarding the statistics see Table 1.

**Table 1:**
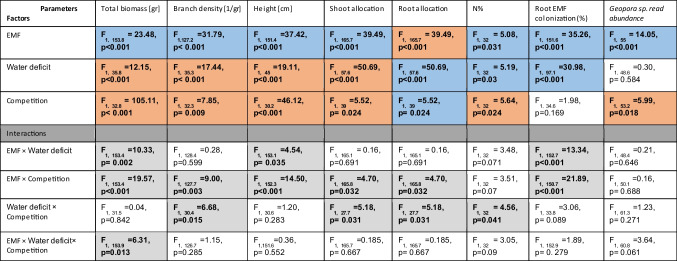
Statistical analysis of pine growth and *Geopora* read abundance according to water, competition and ectomycorrhiza treatments and the interactions between them. The orange color represents a negative correlation between the treatments and the growth index/ *Geopora* read abundance, while the blue color represents a positive correlation. Significant interactions are shaded in grey

### Pine biomass

**Fig. 1:**
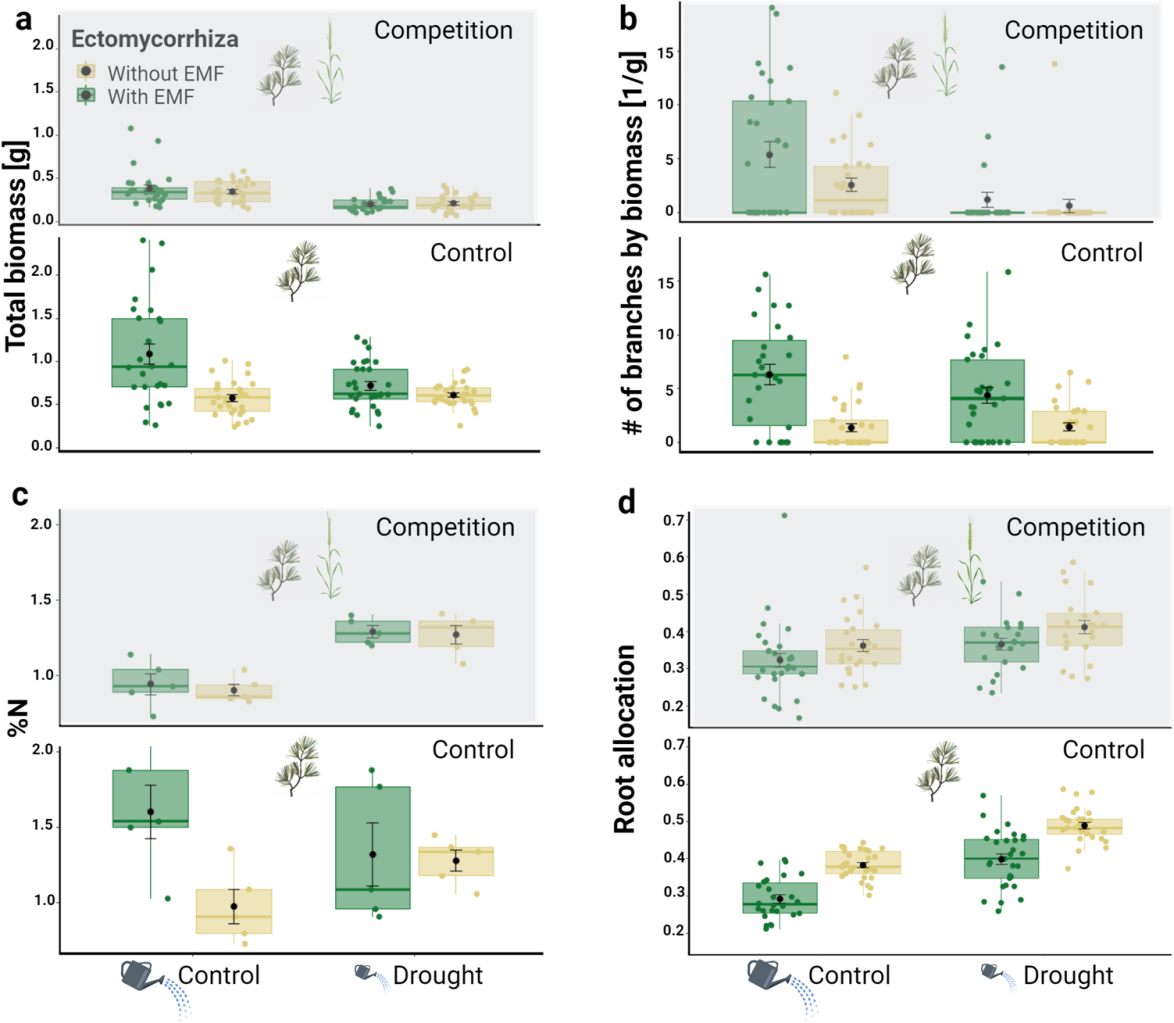
Pine seedling (**a**) total biomass [g], (**b**) number of branches by biomass [1/g], (**c**) needles N% and (**d**) root allocation, divided by water/drought (along the horizontal axis), competition (top and bottom figures in each panel; gray panels represent competition) and ectomycorrhiza treatments (bar color). The first and third hinge of each box plot represent the 25th and 75th percentile, the middle hinge is the median and the black point is the mean ± SE

As expected, drought caused a decrease of 20% to the pine's total biomass (Ample watering: 0.588 ± 0.442 g; Drought: 0.47 ± 0.288 g, Fig. 1a). Similarly, competition caused a decrease of 60% in the pine's total biomass (No-competition: 0.736 ± 0.399 g; Competition: 0.297 ± 0.16 g, Fig. 1a). However, forest soil addition led to an increase of 34% in pine biomass (Non-inoculated: 0.456 ± 0.218 g; Inoculated: 0.61 ± 0.479 g, Fig. 1a). Nevertheless, the positive effect of ectomycorrhiza was more evident when the pines were watered frequently (Fig. 1a). In addition, the positive effect of ectomycorrhiza was more evident when the pines were not facing competition (Fig. 1a). Interestingly, when pines were not experiencing any stress, ectomycorrhiza had a positive effect on the total biomass of the pines. However, when the pines were experiencing either one stress or the combined stress of both drought and competition, ectomycorrhiza did not increase the pines’ total biomass (Fig. 1a). See the supplementary materials for a specific analysis of the effect of grass biomass on pine biomass (Figs. S5 + S6).

### Shoot branching

Like the pine's total biomass, forest soil addition led to an increase of 192% to the pines’ branch density (Non-inoculated: 1.52 ± 2.64 1/g; Inoculated: 4.44 ± 5.14 1/g, Fig. 1b), while drought and competition both caused a decrease of 46% and 19% to the pines branch density (Ample watering: 3.83 ± 4.84 1/g; Drought: 2.06 ± 3.49 1/g, and No-competition: 3.27 ± 4.0 1/g; Competition: 2.66 ± 4.7 1/g, for drought and competition respectively; Fig. 1b). However, competition had a stronger negative effect than drought (Fig. 1b). In addition, the positive effect of ectomycorrhiza was more evident when the pines were not experiencing competition (Fig. 1b).

### Pine height

As expected, drought and competition both caused a decrease of 15% and 25% to the pine's height (Ample watering: 8.17 ± 3.07 cm; Drought: 6.91 ± 2.36 cm, and No-competition: 8.57 ± 3.03 cm; Competition: 6.43 ± 2.04, for water and competition respectively, Fig. S3). Furthermore, forest soil addition led to an increase of 27% in pine height (Non-inoculated: 6.66 ± 1.6; Inoculated: 8.48 ± 3.43, Fig. S3). However, the positive effect of ectomycorrhiza was more evident when the pines were either watered frequently (Fig. S3) or not experiencing competition (Fig. S3).

### Pine nitrogen content

As expected, competition caused a decrease of 15% to the pine's nitrogen content (No-competition: 1.3 ± 0.39 %; Competition: 1.1 ± 0.21 %, Fig. 1c). Drought caused an increase of 14% in the pine's nitrogen content (Ample watering: 1.11 ± 0.375 %; Drought: 1.29 ± 0.24 %, Fig. 1c). Forest soil addition also led to an increase of 14% in pine nitrogen content (Non-inoculated: 1.11 ± 0.23 %; Inoculated: 1.29 ± 0.38 %, Fig. 1c). Nevertheless, the negative effect of competition was only evident when water was not limiting (Fig. 1c).

### Pine root allocation (root mass divided by total biomass)

Drought caused an increase of 12% to the pine's root allocation (Ample watering: 0.34 ± 0.09; Drought: 0.42 ± 0.08, Fig. 1d), while competition and forest soil addition led to a decrease of 8% and 15% to the pine's root allocation (No-competition: 0.39 ± 0.09; Competition: 0.36 ± 0.09, and Non-inoculated: 0.41 ± 0.08; Inoculated: 0.35 ± 0.09, for competition and EMF respectively, Fig. 1d). However, the negative effect of competition was more evident when the pines were under water stress (Fig. 1d). In addition, the negative effect of ectomycorrhiza was more evident when the pines were not experiencing competition (Fig. 1d). Similar yet opposite results were obtained for the pine’s shoot allocation.

### Ectomycorrhizal fungal colonization

#### Root EMF colonization percents

**Fig. 2:**
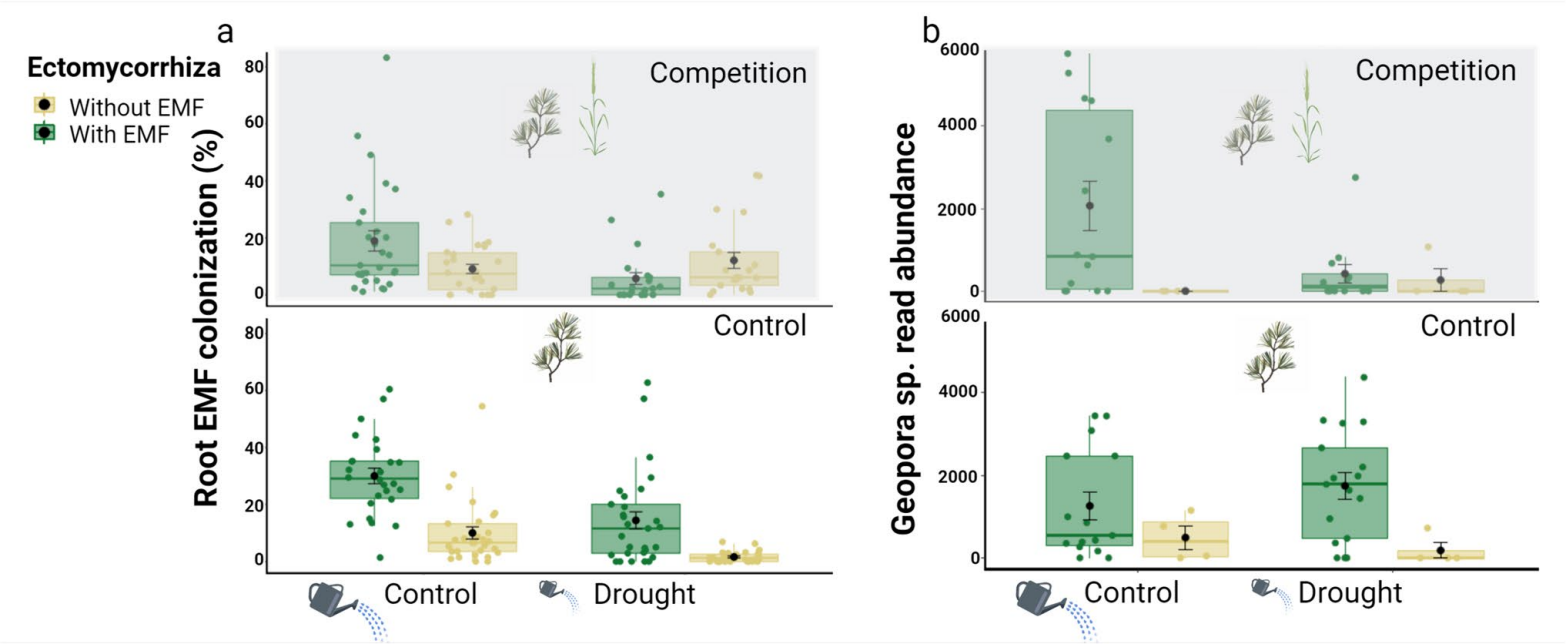
**a** The mean percentage of EMF colonized root-tips and **b** Geopora sp. sequence abundance, according to the water/drought (along the horizontal axis), competition (top and bottom figures in each panel) and ectomycorrhiza treatments (green/ yellow bars for, with/without EMF inoculum, respectively). The first and third hinge of each box plot represents the 25th and 75th percentile, the middle hinge is the median and the black point is the mean ± SE

Drought caused a decrease in root EMF colonization percentages (Ample watering: 17.3 ± 16.6 %; Drought: 8.42 ± 12.4 %, Fig. 2a). Furthermore, the addition of forest soil led to an increase in root EMF colonization percentages (Non-inoculated: 7.9 ± 9.92 %; Inoculated: 18.2 ± 17.9 %, Fig. 2a).

#### *Geopora* sp. read abundance

The inoculated plants were strongly dominated by a single taxon belonging to the genus *Geopora* (99.44%), which had a higher sequence abundance on the roots of plants inoculated with EMF in comparison to non-inoculated plants (Fig. 2b). On average, the sequence abundance of *Geopora* decreased in the presence of a competitor (Fig. 2a). However, this was evident only under drought (**non-significant**; Ectomycorrhiza × Water × Competition: F_1, 60.8_ = 3.64, p = 0.061, Fig. 2b), it appears that when plants were suffering a single stress, *Geopora* was still present on their roots. However, when encountering the combined stress of both drought and competition, *Geopora* sequence abundance was reduced to the same level as in plants that were not inoculated at all. In addition, there was a significant positive correlation between branch density and sequence abundance of *Geopora* (Pearson's r‏ = 0.438, p < 0.001).

## Discussion

We hypothesized that stressful conditions should increase the relative advantage that EMF provide to pine seedlings. Our hypothesis was not supported by the data. Specifically, while forest soil inoculum resulted in increased growth under all conditions, its positive effect was greatly diminished (becoming not significant) under either, each or both stresses. Surprisingly, the shoot branching pattern of the seedlings showed a qualitatively different response to inoculation compared to those of the seedling height and mass.

We found a discrepancy between EMF presence and their effect on seedling growth. While EMF were present and beneficial (a 202% increase in biomass) under benign conditions, under each of the single stresses no apparent enhancement was observed in pine growth, even though EMF presence was maintained (Figs. 1a, 2). The preservation of the interaction even when it does not seem to benefit pine growth is somewhat surprising. This apparently altruistic pattern can be the result of unmeasured benefits such as pathogen resistance (Gonthier et al. 2019). The fact that the fungi sustain on the roots under harsh conditions could also provide an advantage in case conditions improve. Furthermore, inconsistent with our hypothesis, under the combined stress, the seedlings demonstrated both reduced growth and a reduction in EMF presence (to levels similar to those of the uninoculated pines). We interpret this to mean that under single stress conditions, the fungi in our experiment were able to survive but were unable to provide growth benefits to the seedlings. When both stressors occurred simultaneously, it seems as if the fungi’s mere survival was hampered.

Our results indicate that the presence of EMF was positively correlated with N content only under non-stressful conditions (Fig. 1c). The fact that under competition and ample watering EMF was abundant but did not have a positive effect on pine biomass (Figs. 1a, 2), suggests that the mere presence of *Geopora* is not sufficient to enhance pine growth (Fig. 2). This complexity might be related to the soil organic matter available for EMF decomposition (Shah et al. 2016; Nicolás et al. 2019). In light of our findings, we further hypothesize that the available inorganic minerals in the soil were quickly uptaken by the grass, as shown in another study (Cheng and Bledsoe 2004) therefore limiting the amount of resources avialable for the pine seedling and resulting in low N content in the needles (Fig. 1c). Although soil organic matter can be a nutrient source for some EMF fungi (Phillips et al. 2014), *Geopora* which is part of ascomycota group, probably lacks the ability to harvest nutrients out of soil organic matter (Lundeberg 1970; Read et al. 1989; Pellitier and Zak 2018). Future studies manipulating EMF, competition and soil organic matter under greenhouse and field conditions could shed more light on this complex interaction.

Unlike the positive effects of ectomycorrhiza on biomass, which disappeared under a single stress, the effect on shoot branching was maintained under either competition or drought but not under both (Fig. 1b). This positive effect on shoot branching was evident even after controlling for pine biomass. The optimal branching strategy is expected to depend on ecological conditions such as the competing plant community, light availability and the species biology and strategies (Evers et al. 2011; Gruntman et al. 2017; MacFarlane and Kane 2017). A similar effect was found in redwood seedlings inoculated with arbuscular mycorrhizal fungi (Willing 2019) and in a previous study with Aleppo pine seedlings (Livne-Luzon et al. 2021).

Interestingly, while the growth effects of EMF diminished under a single stress, the shoot branching effect was maintained and was correlated with fungal abundance. Therefore, the mechanism linking EMF and shoot branching seems to function independently of the known growth effects of EMF. We suggest that the shoot branching mechanism is dependent on the mere presence of the fungi, which might induce a hormonal effect on the shoot branching pattern. For example, production of the growth hormone auxin was connected with EMF colonization in poplar (Felten et al. 2009), and cypress saplings exuded auxin when inoculated with rhizosphere bacteria (Oppenheimer-Shaanan et al. 2022). Moreover, an increase in ABA hormone, which is related to drought tolerance and is known to affect branching, was seen in redwood seedlings colonized by AMF under drought (Willing 2019). This proposed mechanism should be tested under different ecological scenarios, to better understand the role of the fungi in changing the shoot structure and the consequences of this change to plant adaptation to stress.

Quantifying the colonization of EMF is a challenging task, even more so, when considering relatively understudied species, without a known distinct morphology (Janowski and Leski 2023). As expected, during root tip collection we did not observe colonized root-tips on plants that were not amended with EMF inoculum. However, some levels of colonization were revealed by sequencing. These low levels of read abundance could be the result of either minute amounts of inoculum that were present in the potting material or of a sequencing spillover. Nevertheless, the reduced growth of these plants suggests that these levels of colonization are not ecologically significant. In our specific study, more than 99.4% of the reads in all EMF samples were of a single genus (*Geopora*). This high dominance of *Geopora* in young *Pinus halepensis* seedlings has been shown to occur in previous studies (Livne-Luzon et al. 2016, Livne-Luzon et al. 2017, Livne-Luzon et al. 2021). We suggest that under effectively single-taxon samples, PCR amplification biases should be negligible, making read abundance a possible proxy for the comparison of this taxon’s abundance among samples. It appears that when pines were suffering a single stress, the abundance of *Geopora* on the pines’ roots was not reduced (Fig. 2). However, when encountering the combined stress of both drought and competition, *Geopora* sequence abundance was reduced to the same level as in pines that were not inoculated at all.

Although our study provides critical insights into the interplay between drought and inter-plant competition, it's imperative to recognize the constraints inherent in greenhouse-based research. Such controlled settings may not entirely capture the multifaceted environmental interactions prevalent in natural ecosystems. Nonetheless, this approach is indispensable for its heightened internal validity, allowing for detailed manipulation and observation of specific factors. Our choice to employ natural soil inoculums, aimed at approximating ecological conditions more closely, is not without its challenges. The decision to add natural forest soil limits the internal validity of the study by increasing the number of alternative explanations regarding the obtained results. We find the effect of other soil microbiota in the inoculum to be the main concern. However, we interpret the change in root allocation (a known effect of EMF; (Smith and Read 2010) and the effect of inoculation on EMF read abundance (Fig. 2b) as strong indicators of EMF as the main drivers of the discovered effects. In light of the above, we find natural inoculum to be preferred over a species poor synthetic community.

**Fig. 3:**
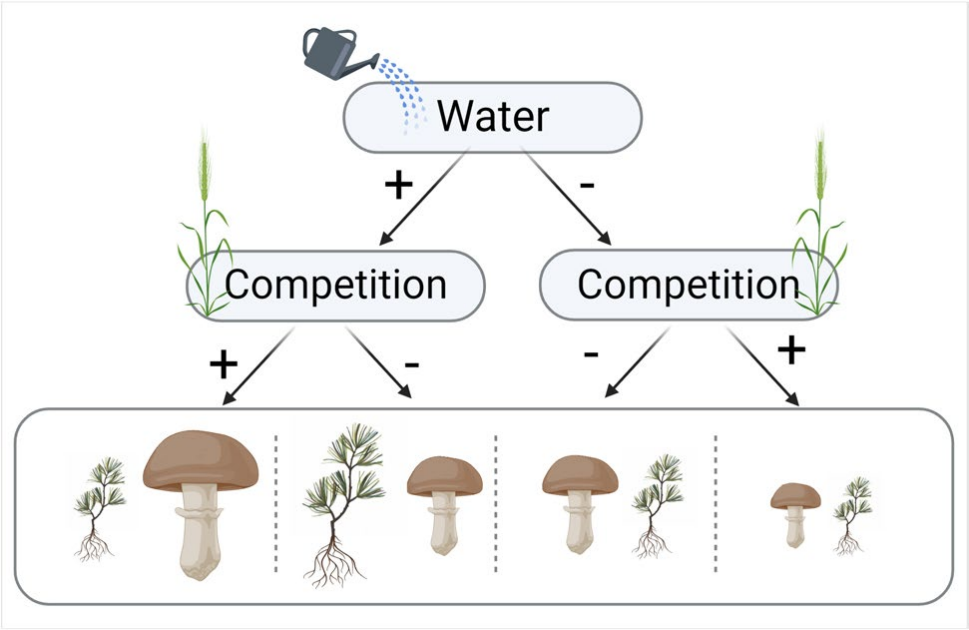
A conceptual hierarchy of the effect of both water and competition stress on the EM relationship. The size of the fungi and the seedling represent the approximate amount of *Geopora* sp. sequence abundance and the seedlings total biomass respectively in the water/competition treatments

In conclusion, while the role of EMF on plant growth under benign conditions is well established, less is known regarding its role under stress. In our study, under single or combined stressors, colonization by *Geopora* did not contribute to the pines' total biomass. Nonetheless, when pines were experiencing a single stress, EMF presence still increased their branch number. Furthermore, pines experiencing drought might be limited in their ability to support their mycorrhizal partners because of their limited ability to photosynthesize (Parke et al. 1983), however such responses might be species dependent On the other hand, plants experiencing competition might rely on their mycorrhizal partners to decompose organic matter and alleviate nutrient stress caused by their competitors and should be more engaged with EMF (Peay 2016; Van Nuland et al. 2023). The role of EMF in elevating drought and competition stresses of establishing seedlings seems to be complex (Fig. 3) and further studies, especially about inter-plant competition, are needed. It would therefore be interesting to study the influence of EMF communities that originate from habitats differing in their aridity. Deciphering the role of EMF in seedling establishment under drought and competition could help us better predict forest dynamics, including reforestation, under current global changes.

### Supplementary Information

Below is the link to the electronic supplementary material.

Supplementary file1 (DOCX 1180 KB)

## Data Availability

Sequences are submitted to the National Center for Biotechnology Information Sequence Read Archive with the accession codes: Bioproject PRJNA1111766.
