# Supplementary material for: Context-dependent benefits of forest soil addition on Aleppo pine seedling performance under drought and grass competition
Source: Mycorrhiza. 2024 May 18;34(3):217–27. doi: 10.1007/s00572-024-01151-x (PMC11166812; doi:10.1007/s00572-024-01151-x)
Supplement: Supplementary file 1 — Supplementary file1 (DOCX 1180 KB) [file 572_2024_1151_MOESM1_ESM.docx]

**Electronic Supplementary Material**

Table S1: Forest soil locations

| Location |  |
| --- | --- |
| 1 | (N 35°33'49"E 59°33'13") |
| 2 | (N 35°33'34"E 24°33'14") |
| 3 | (N 35°34'24"E 46°33'14") |
| 4 | "(N 35°33'07"E 29°33'13") |

| Average of the weight of the pots | Amount of water (ml) | Date |
| --- | --- | --- |
| 4.72±0.24 | 400 | 7.5.2020 |
| 4.75±0.44 | 400 | 12.5.2020 |
| 4.637±0.21 | 400 | 14.5.2020 |
| 4.61±0.22 | 400 | 18.5.2020 |
| 4.92±1.02 | 400 | 21.5.2020 |
| 4.66±0.2 | 400 | 26.5.2020 |
| 4.7±0.21 | 400 | 31.5.2020 |
| 4.69±0.22 | 400 | 4.6.2020 |
| 4.66±0.2 | 400 | 8.6.2020 |
| 4.73±0.22 | 400 | 14.6.2020 |
| 4.69±0.21 | 200 | 18.6.2020 |
| 4.65±0.2 | 400 | 23.6.2020 |
| 4.7±0.23 | 200 | 28.6.2020 |
| 4.69±0.23 | 200 | 1.7.2020 |
| 4.67±0.23 | 200 | 5.7.2020 |
| 4.7±0.24 | 200 | 9.7.2020 |
| 4.71±0.23 | 200 | 13.7.2020 |
| 4.72±0.24 | 200 | 17.7.2020 |
| 4.68±0.23 | 200 | 22.7.2020 |
| 4.67±0.23 | 200 | 26.7.2020 |
| 4.69±0.22 | 100 | 2.8.2020 |
| 4.65±0.22 | 100 | 6.8.2020 |
| 4.62±0.21 | 200 | 11.8.2020 |
| Average= 4.69±0.06 | Average= 286.96±111.53 | Average time gap= 4.36±1.02 |

Table S2: Manual irrigation details

Table S3: Statistical analysis of plant growth according to water, competition and ectomycorrhiza treatments and the interactions between them with outliers. The orange color represents a negative connection between the treatments and the growth index/ EMF present, while the blue color represents a positive connection.


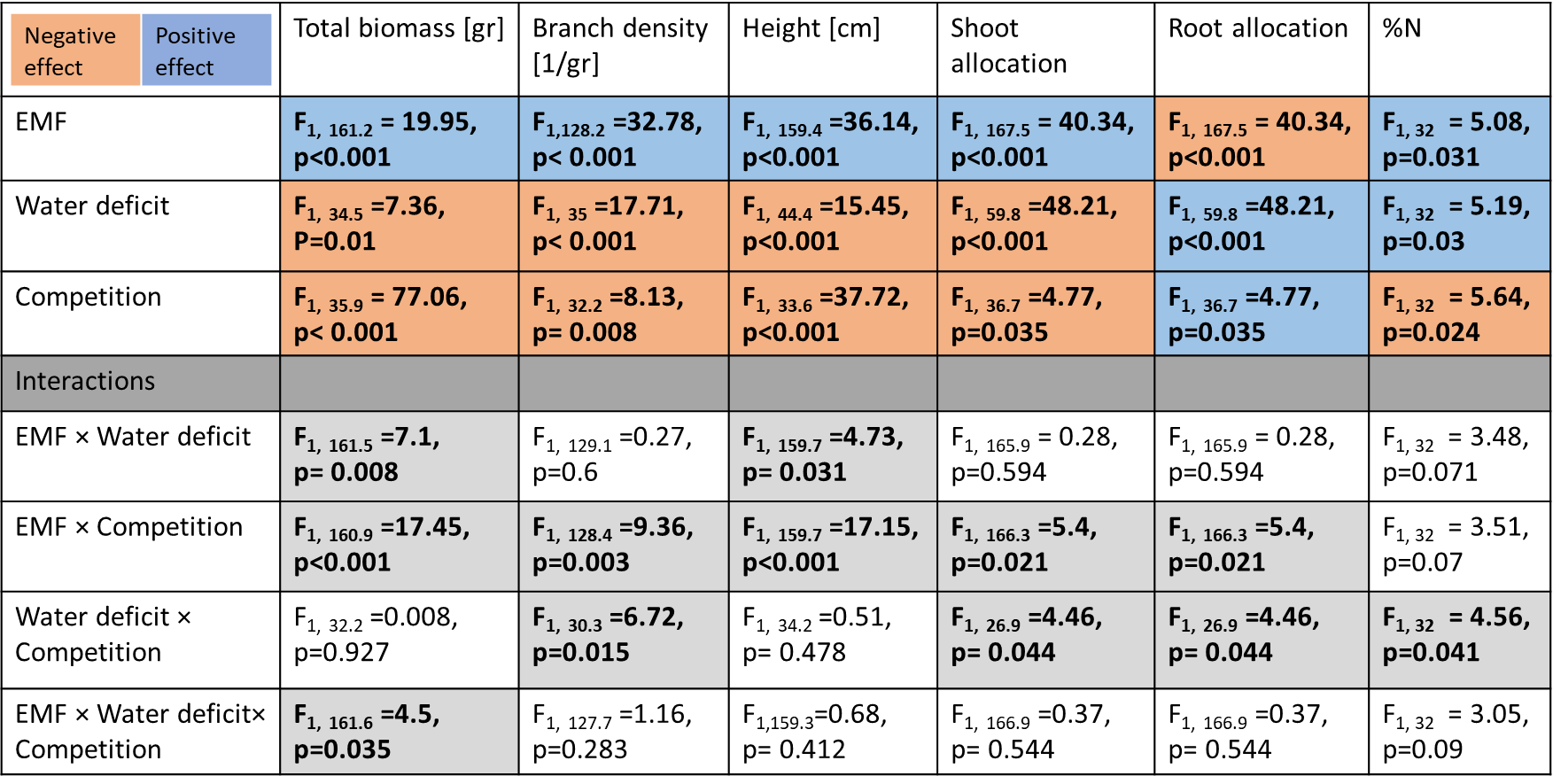


| - | + | - | + | Competition  \  Drought |
| --- | --- | --- | --- | --- |
| 17  Drought stress | 13  Double stress | 4  Drought stress | 4  Double stress | + |
| 16  Optimal condition | 14  Competition stress | 4  Optimal condition | 4  Competition stress | - |
| With EMF presence | With EMF presence | No EMF presence | No EMF presence | EMF |

Table S4: Number of samples for molecular identification of fungal species‏

Table S5: Taxonomic Distribution and ITS2 Sequence Abundance in root-associated fungal Communities.

This table summarizes the taxonomic classification and total read abundance of fungal taxa identified associated with individual seedlings. The read abundance values represent the cumulative count of ITS2 sequence reads for each taxon across all sampled seedlings, obtained via pooled ITS2 amplicon sequencing. Taxonomic assignments were determined by referencing ITS2 sequences against the UNITE database.

| Phylum | Class | Order | Family | Genus | Species | Accession number | Total read abundance | % of total read abundance |
| --- | --- | --- | --- | --- | --- | --- | --- | --- |
| Ascomycota | Pezizomycetes | Pezizales | Pyronemataceae | Geopora | NA | HE687048 | 87357 | 99.441 |
| Ascomycota | Pezizomycetes | Pezizales | Tuberaceae | Tuber | oligospermum | KM247652 | 382 | 0.435 |
| Ascomycota | Pezizomycetes | Pezizales | Tuberaceae | Tuber | nitidum | NR_174649 | 55 | 0.063 |
| Ascomycota | Sordariomycetes | Branch06 | NA | NA | NA | MT278027 | 15 | 0.017 |
| Ascomycota | Pezizomycetes | Pezizales | Tuberaceae | Tuber | NA | HE687179 | 10 | 0.011 |
| Basidiomycota | Agaricomycetes | Atheliales | Atheliaceae | Amphinema | byssoides | JN943914 | 7 | 0.008 |
| Ascomycota | Pezizomycetes | Pezizales | Tuberaceae | Tuber | puberulum | KX354290 | 7 | 0.008 |
| Ascomycota | Pezizomycetes | Pezizales | Pyronemataceae | Sphaerosporella | brunnea | MT156500 | 6 | 0.007 |
| Basidiomycota | Agaricomycetes | Thelephorales | Thelephoraceae | Tomentella | coerulea | HE687136 | 5 | 0.006 |
| Ascomycota | Pezizomycetes | Pezizales | Pyronemataceae | Pustularia | NA | MT236609 | 2 | 0.002 |
| Basidiomycota | Agaricomycetes | Thelephorales | Thelephoraceae | Tomentella | coerulea | JX316246 | 1 | 0.001 |
| Basidiomycota | Agaricomycetes | Russulales | Russulaceae | Russula | densifolia | MH930947 | 1 | 0.001 |


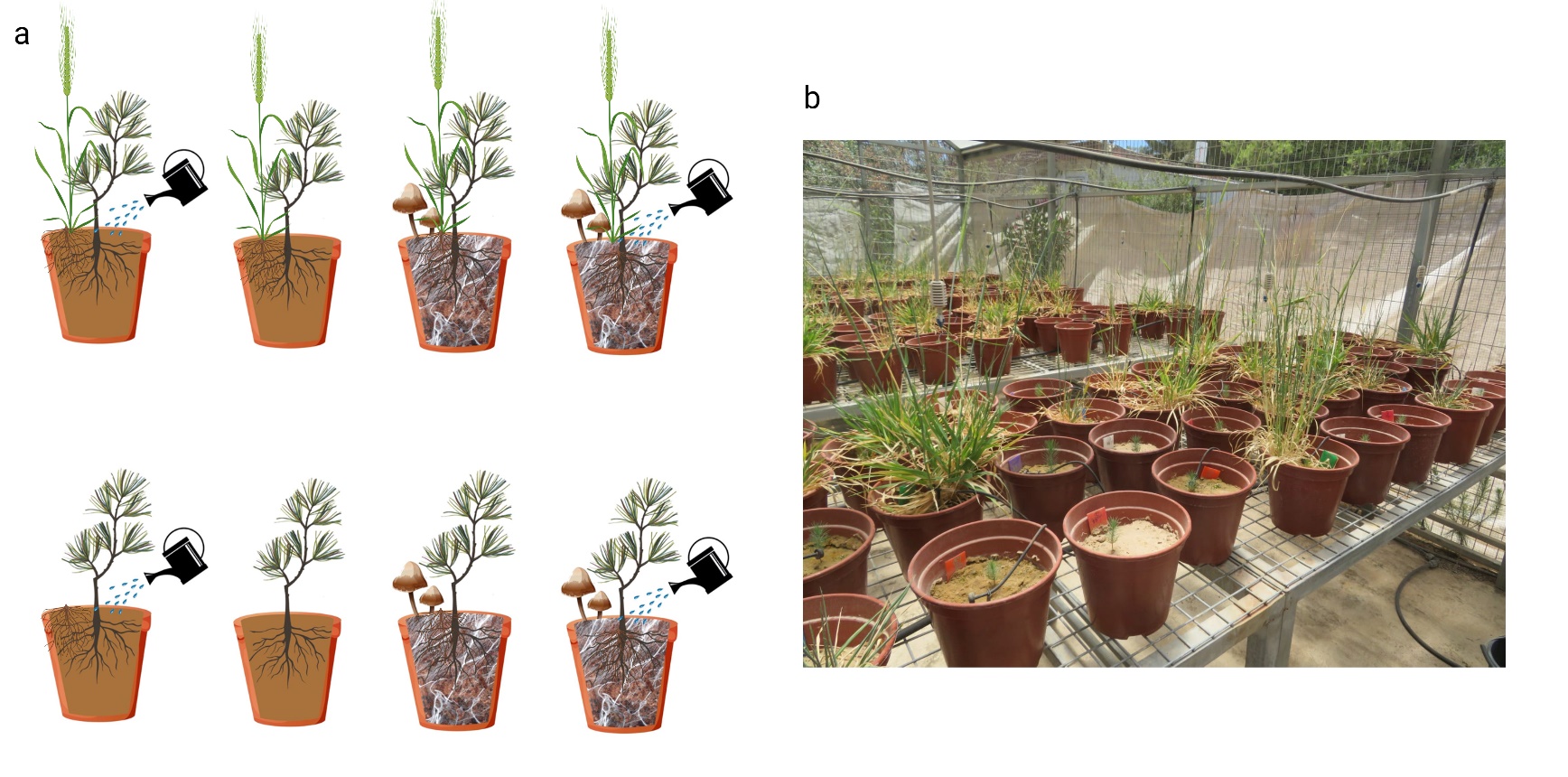


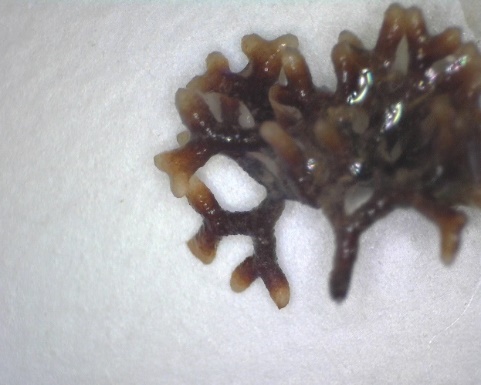


c

Fig. S1: Illustration of the Experimental Design. (a) Represents the eight treatments, and (b) displays a photograph of the experiment after a few weeks of growth. (c) typical mycorrhizal root tip morphology later identified as colonized by *Geopora* sp.


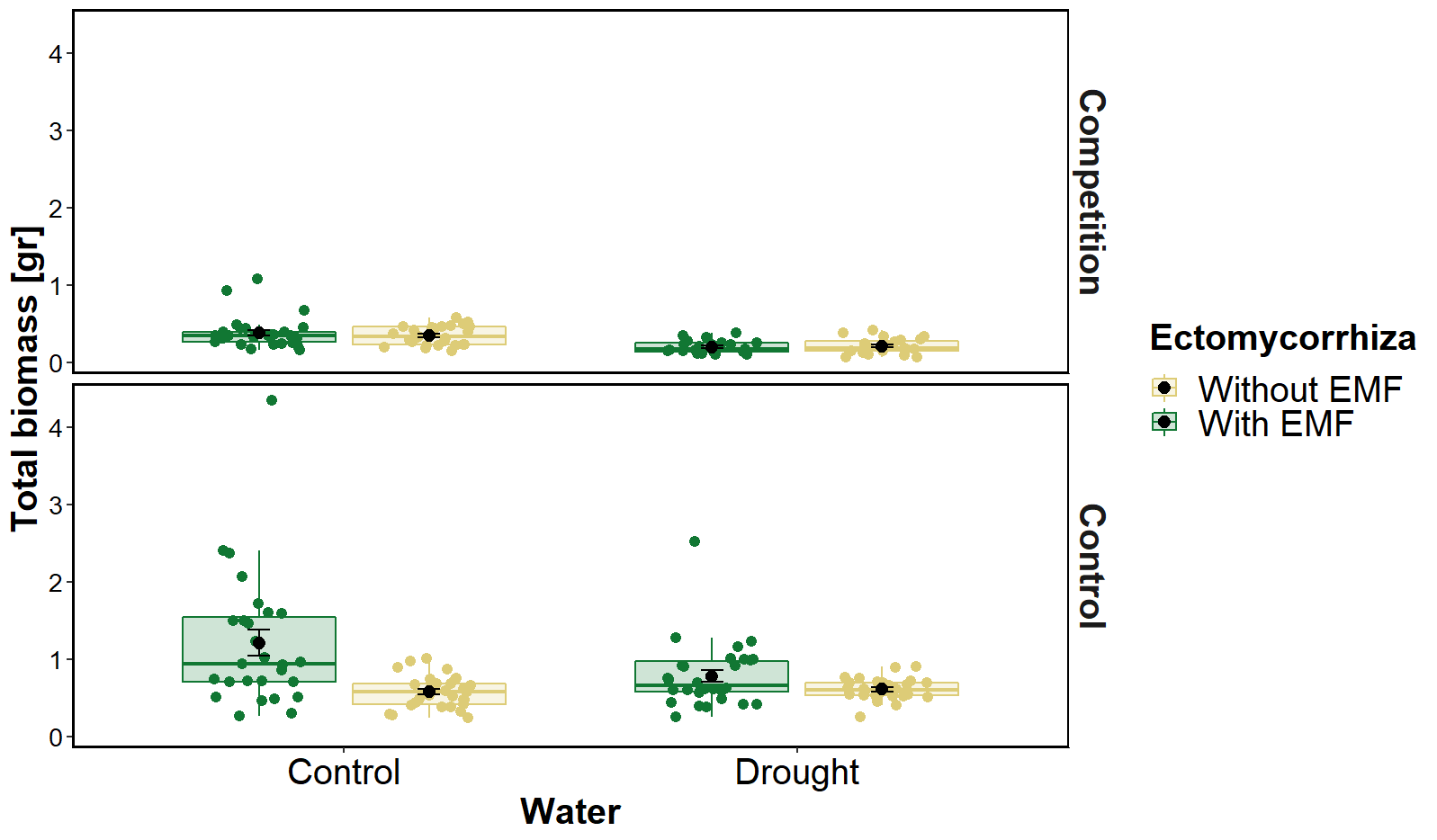
Fig.S2 Pine seedling total biomass [g] according to water, competition and ectomycorrhiza treatments with the two outliers (circled). The first and third hinge of each box plot represent the 25th and 75th percentile, the middle hinge is the median and the black point is the mean ±SE


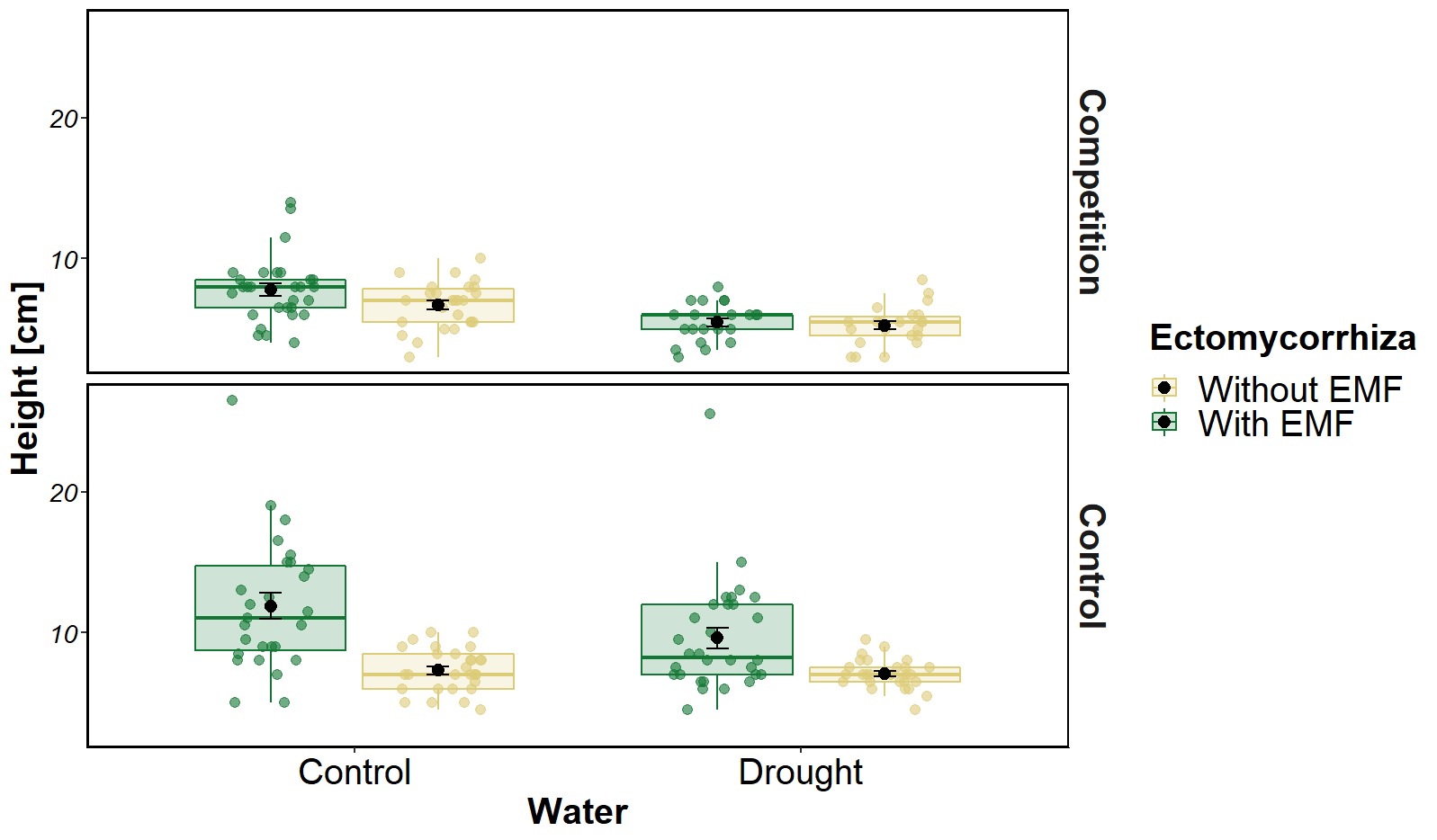
Fig.S3 Pine seedling height according to water, competition and ectomycorrhiza treatments with the two outliers (circled). The first and third hinge of each box plot represent the 25th and 75th percentile, the middle hinge is the median and the black point is the mean ±SE


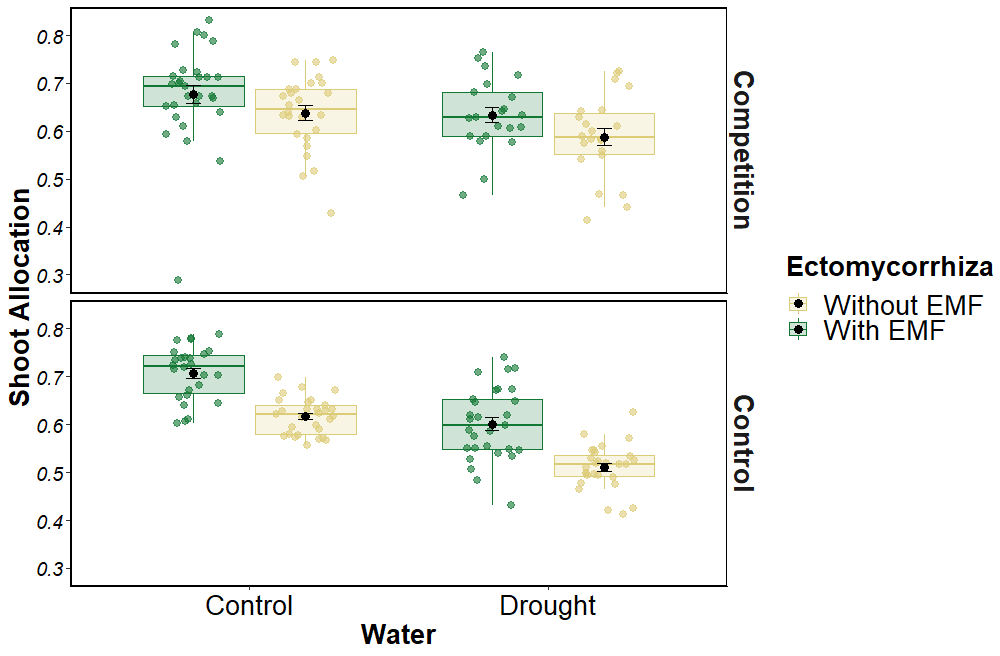


Fig.S4 Pine seedling shoot allocation according to water, competition and ectomycorrhiza treatments. The first and third hinge of each box plot represent the 25th and 75th percentile, the middle hinge is the median and the black point is the mean ±SE

**The relation between pine and *Hordeum Spontaneous* biomasses**:

We used a general linear mixed model, using a fully factorial design, to account for differences in the plant's total biomasses. The following explanatory variables were included as fixed factors: water treatment and *Hordeum Spontaneous* biomass, as well as their interaction. The experimental blocks were included in the model while allowing for random slopes and intercepts for each fixed factor.

As expected, drought caused a decrease of 43% to the plant's total biomass (Ample watering: 0.366±0.169; Drought: 0.208±0.089; F_1,93.2_ =12.5, p<0.001, Fig.S5). Notice the 4 conspicuous outliers.


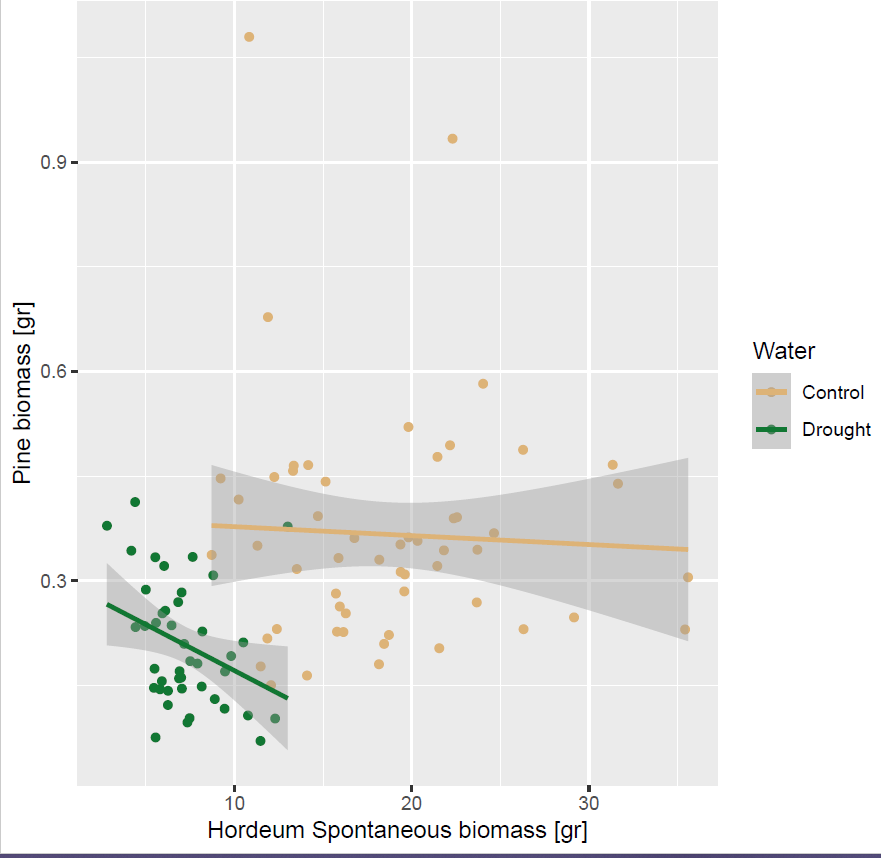


Fig. S5: Pine seedling total biomass [g] according to water treatment and Hordeum Spontaneous biomass with outliers.

We here report the analysis after the removal of the 4 outliers. As expected, drought caused a decrease of 39% to the plant's total biomass (Ample watering: 0.335±0.105; Drought: 0.204±0.086; F _1,89.7_ =26.81, p<0.001, Fig.S6). Similarly, the size of the grass competitor caused a decrease in the plant's total biomass (F _1, 87.2_ = 7.63, p=0.007, Fig.S6). Nevertheless, the negative effect of high competitor biomass was only evident when the plants were grown under water stress (Water × Hordeum Spontaneous biomass: F _1, 90_ =10.19, p=0.002, Fig.S6).


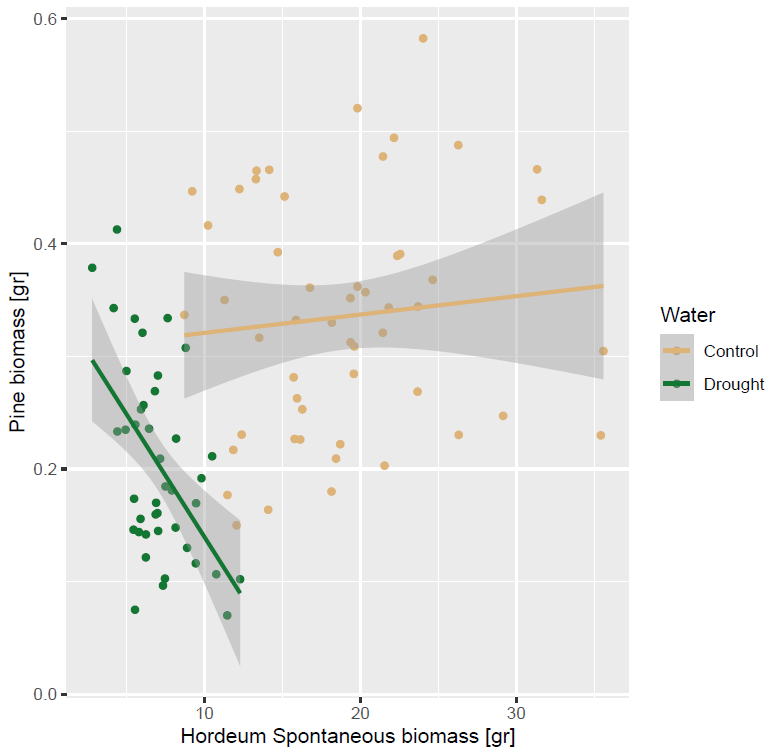


Fig. S6: Pine seedling total biomass according to water treatment and *Hordeum Spontaneous* biomass without outliers.

Interpretation

The effect of the size of the grass competitor (estimated by grass biomass) on the biomass of the pine seedling was dependent on water availability (interaction grass X drought only after removal of outliers). While the presence of a competitor always had a negative effect on pine biomass (Fig. 1a) the size of the competitor seems to have a negative effect only under drought conditions (pines experiencing both stresses simultaneously, Fig. S6). Grass plants under high water availability might have been large enough to monopolize the soil resources (besides water) regardless of their size. The grass plants under drought were significantly smaller and might have not been able to take up all the soil resources. The ability of grass plants under drought to uptake resources could have been proportional to their size.
